# Rizatriptan in migraineurs with unilateral cranial autonomic symptoms: a double-blind trial

**DOI:** 10.1007/s10194-012-0440-y

**Published:** 2012-03-30

**Authors:** Piero Barbanti, Luisa Fofi, Valentina Dall’Armi, Cinzia Aurilia, Gabriella Egeo, Nicola Vanacore, Stefano Bonassi

**Affiliations:** 1 Headache and Pain Unit, Department of Neurological, Motor and Sensorial Sciences, IRCCS San Raffaele Pisana, Via della Pisana 235, 00163 Rome, Italy; 2 Unit of Clinical and Molecular Epidemiology, IRCCS San Raffaele Pisana, Via della Pisana 235, 00163 Rome, Italy; 3 National Center of Epidemiology, National Institute of Health, Rome, Italy

**Keywords:** Migraine, Cranial autonomic symptoms, Trigemino-autonomic reflex, Treatment, Rizatriptan

## Abstract

The objective and background is to confirm in a double-blind, placebo-controlled study the high triptan response rates we had previously reported in an open study in migraine patients with unilateral cranial autonomic symptoms. In this randomized, double-blind, placebo-controlled study 80 migraineurs with unilateral cranial autonomic symptoms were assigned to receive rizatriptan 10 mg wafer or placebo (ratio 1:1) and treated for a single moderate or severe migraine attack. The primary endpoints were pain freedom at 2 h and total migraine freedom at 2 h. Secondary endpoints included pain relief, no associated symptoms and sustained pain freedom or relief. Significantly more patients reported pain freedom at 2 h after taking rizatriptan (54 %) than after placebo (8 %) (therapeutic gain 46 % [28 %; 64 %]; *P* < 0.001). Similarly, significantly more patients reported total migraine freedom at 2 h after rizatriptan (51 %) than after placebo (8 %) (therapeutic gain 43 % [26 %; 61 %]; *P* < 0.001). Rizatriptan was also more effective than placebo on most secondary endpoints. We confirm in a placebo-controlled study our previous data suggesting that the presence of unilateral cranial autonomic symptoms in migraineurs predicts a positive response to triptans, probably owing to intense trigeminal peripheral afferent activation which strongly recruits peripheral neurovascular 5-HT1B/1D receptors. Acute and preventive pharmacological trials in migraine should focus also on this subset of migraine patients.

## Introduction

Migraine pain depends on trigeminovascular system activation that induces vasoactive neuropeptide release from trigeminal perivascular axons leading to neurogenic inflammation that stimulates meningeal sensory fibers and transmits nociceptive information centrally, along the trigeminal axons, to the trigeminal nucleus caudalis, and from there rostrally to the thalamus and cortex [1]. In some migraineurs, activating the trigeminovascular system may trigger the efferent parasympathetic arm of the trigemino-autonomic reflex [2]. In these migraineurs, whose prevalence ranges from 26.4 % in the general migraine population to 45.8 % in patients attending a Headache Center, the clinical hallmarks are unilateral cranial autonomic symptoms (UAs) such as conjunctival injection, lacrimation, nasal congestion/rhinorrhea, ptosis, eyelid swelling or forehead/facial sweating, singly or combined. Migraine headache is usually more strictly unilateral and more severe in patients with UAs than in the general migraine population [3, 4].

In an open study with sumatriptan 50 mg, we previously suggested that UAs in migraineurs may predict a positive response to triptans [5]. Their possible predictive value received further support from a study describing detectable serum vasoactive intestinal polypeptide (VIP), the biochemical marker of parasympathetic activation, in the external jugular blood in one-half of migraine patients who responded to rizatriptan [6]. The complex yet clinically important issue of whether UAs predict triptan responses in migraine therefore awaits confirmatory data from a placebo-controlled study. Having this information would allow more tailored therapy for treating acute migraine.

In this study, to find out more about triptan response rates in patients with UAs, in a randomized, double-blind, placebo-controlled, parallel-group study we used oral rizatriptan 10 mg, one of the most commonly used and effective triptans [7], for acute therapy in a consecutive series of patients with migraine and UAs. To do so we chose stringent primary outcome measures for treatment efficacy including pain and total migraine freedom at 2 h [8]. We also ensured that patients waited before taking oral rizatriptan until their headache became moderate to severe.

## Methods

### Study population and design

For this randomized, double-blind, placebo-controlled, parallel-group, outpatient study to assess the efficacy of rizatriptan 10 mg wafer in treating a single acute migraine attack in migraineurs with UAs, patients were consecutively recruited from our Headache and Pain Unit. Patients were eligible for the study if they were ≥18 years of age, had a history of migraine with or without aura for at least 1 year, and in the 2 months before screening had experienced 1–8 moderate or severe migraine attacks per month [9]. Patients taking migraine prevention medication were allowed to enter the study if their prescribed daily dose had remained unchanged during the 3 months before screening. Patients taking propranolol, methysergide, serotonin norepinephrine reuptake inhibitors, selective serotonin reuptake inhibitors, or monoamine oxidase inhibitors within 14 months of the screening visit were not eligible. Patients with history or clinical evidence of ischemic heart disease or symptoms or findings consistent with ischemic heart disease, coronary artery vasospasm, or other significant underlying cardiovascular disease and those with clinical, laboratory, or electrocardiographic evidence of uncontrolled hypertension, uncontrolled diabetes, or significant pulmonary, renal, hepatic, endocrine, or other systemic disease were also excluded.

Patients attended the hospital for a screening visit to assess eligibility and undertake physical examinations. The interview determined whether patients experienced UAs by asking the following question: “During the migraine attack do you also have *at least one* of the following symptoms: unilateral conjunctival injection, lacrimation, nasal congestion/rhinorrhea, ptosis, eyelid swelling or forehead/facial sweating?” [3]. Patients who met all the study entry criteria were enrolled and randomly allocated to receive either rizatriptan 10 mg wafer or placebo (ratio 1:1). Patients were encouraged to take migraine medication as soon as their migraine headache became moderate or severe. If the moderate or severe migraine headache persisted 2 h after dosing, or recurred within 24 h, patients had the option of taking their own rescue medication but triptans and ergot derivatives were prohibited for 24 h after study medication intake.

This protocol was approved by the institutional review board at San Raffaele Pisana Scientific Institute and have therefore been performed in accordance with the ethical standards laid down in the 1964 Declaration of Helsinki. All patients gave their informed consent prior to their inclusion in the study.

### Data collection

During the 24 h after taking the initial dose of study medication, patients recorded subjective assessments of pain severity, presence or absence of associated symptoms, use of rescue medication, and the onset of, if applicable, headache recurrence at specified time points in a paper migraine diary. Subjective adverse experiences were recorded in the diary and rated as mild, moderate or severe. Patients were asked to return to the study site as soon as possible and >7 days after treatment to allow physicians to review the diary, assess medication compliance and monitor tolerability. Headache severity was recorded using a four-grade scale (no pain, mild pain, moderate pain, severe pain) at six time points, baseline (time of taking study drug) and at 0.5, 1, 1.5, 2, and 24 h thereafter. The presence or absence of associated symptoms (nausea, vomiting, photophobia, or phonophobia) was recorded at the same time points as the headache severity ratings. For those patients who had pain relief (pain reduction to mild or none) or pain freedom (no pain) at 2 h, another variable recorded was the presence or absence of headache worsening (recurrence) within 2–24 h. In all patients we also recorded use of rescue medication within 24 h. Tolerability and safety were assessed by asking patients to report spontaneous adverse events (AEs).

### Outcome measures for efficacy

The primary endpoints were pain freedom at 2 h and total migraine freedom (pain freedom and absence of associated symptoms) at 2 h. Secondary endpoints were pain freedom at 0.5, 1 and 1.5 h, pain relief at 0.5, 1, 1.5 and 2 h; absence of nausea, photophobia and phonophobia at 0.5, 1, 1.5 and 2 h; 2–24 h sustained pain relief (pain relief from 2 to 24 h without rescue medication); and 2–24 h sustained pain freedom (pain freedom from 2 to 24 h without rescue medication).

### Randomization sequence generation

The random allocation sequence, including details of any restrictions was produced by Computer Generated Masked Allocation Schedule, Blocking Factor: 4. The allocation sequence was generated by the Pharmaceutical Research and Development Labeling System, Merck & Co., USA. Numbered containers were used to implement the random allocation sequence. The sequence was concealed until unblinding was necessary. The principal investigator assigned participants to the groups, following the masked allocation schedule numbers. All the participants, those administering the treatments and those assessing the outcomes were blinded to group assignment. The success of blinding was guaranteed using similar shaped containers and tablets and by the sealed masked allocation schedule.

### Sample size calculation

**Fig. 1:**
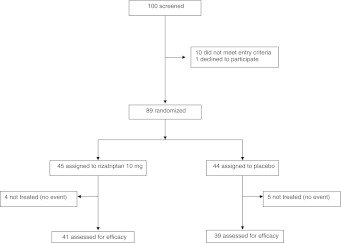
Flow-chart of patients

Sample size was calculated assuming that the therapeutic gain over placebo for migraineurs with UAs treated with rizatriptan 10 mg is similar to that reported in the general migraine population, i.e., 31 % pain freedom at 2 h and 33 % pain relief at 2 h [10]. Given the limited published evidence assessing the effect of rizatriptan 10 mg on the rate of total migraine freedom, the number of patients to be recruited was estimated according to the effect of rizatriptan 10 mg on pain freedom at 2 h. Assuming a type I error rate of 0.05 %, a statistical power of 90 %, and a pain freedom rate at 2 h of 41 % in the treated group versus 10 % in the placebo group [10], we estimated that the study would require a minimum number of 78 patients (39 to be treated with rizatriptan 10 mg and 39 with placebo). A total number of 100 patients was considered for inclusion in the study (Fig. 1).

### Statistical analysis

The presence of heterogeneity between the two treatment groups according to demographic and baseline characteristics was assessed using the unpaired *t* test for continuous variables, and the Pearson’s Chi-square test (or Fisher’s exact test with expected cell frequencies <5 %) for categorical variables. A binomial regression analysis was run to test the effect of rizatriptan 10 mg versus placebo, and the therapeutic gain (defined as the change in outcome induced by rizatriptan 10 mg minus the placebo effect) with its relative 95 % confidence interval (CI), was estimated for all outcome measures. The absence of nausea, phonophobia and photophobia after treatment was analyzed in a subset of individuals who experienced these symptoms at baseline (when the attack began).

To remove the effect of rescue medication, data were analyzed after excluding patients who took a rescue medication before the scheduled time point of outcome measurement. A *P* value <0.05 was the threshold for statistical significance. To take into account the type I error inflation generated by multiple comparisons, the Bonferroni correction was applied (new threshold for type I error <5 %: *P* < 0.0019).

STATA/SE V10 was used for all statistical analyses.

## Results

Of the 100 outpatients screened, 10 failed to meet all the inclusion criteria, 1 declined to participate and 89 were randomly assigned to rizatriptan 10 mg wafer (45 patients) or placebo (44 patients). Four patients in the rizatriptan group and 5 patients in the placebo group were excluded from the efficacy assessment because they lacked a qualifying event (i.e., moderately or severely intense migraine attack) (Fig. 1). Demographic features and baseline characteristics were similar in the active drug and placebo groups (Table 1).

**Table 1:** Socio-demographic and clinical characteristics of migraine patients with unilateral cranial autonomic symptoms

	Rizatriptan 10 mg (41 patients)	Placebo (39 patients)	*P* values
Sex			0.417
Female	33 (80 %)	34 (87 %)	
Male	8 (20 %)	5 (13 %)	
Age (years)	43.95 ± 12.24	41.41 ± 11.70	0.349
Body mass index	22.49 ± 3.03	23.13 ± 3.25	0.364
Illness duration (years)	27.93 ± 15.39	23.42 ± 13.87	0.186
Family history of migraine	34 (82.9 %)	27 (69.2 %)	0.150
MwA	39 (95.1 %)	36 (92.3 %)	0.603
MwA + MA	2 (4.9 %)	3 (7.7 %)	0.603
Attack/month	5.68 ± 3.63	7.18 ± 5.11	0.135
Attack duration (h)			0.650
≤24	20 (48.8 %)	21 (53.8 %)	
>24	21 (51.2 %)	18 (46.2 %)	
Pain location			0.488
Unilateral, alternating side	12 (29.3 %)	7 (18 %)	
Unilateral, same side	25 (61 %)	28 (71.8 %)	
Bilateral	4 (9.8 %)	4 (10.3 %)	
Pain quality			0.298
Pulsating	29 (70.7 %)	24 (61.5 %)	
Pressing	5 (12.2 %)	10 (25.7 %)	
Other	7 (17.1 %)	5 (12.8 %)	
Pain intensity			0.695
Moderate	29 (70.7 %)	26 (66.7 %)	
Severe	12 (29.3 %)	13 (33.3 %)	
UAs (*n*)			0.471
1	25 (62.5 %)	26 (70.3 %)	
>1	15 (37.5 %)	11 (29.7 %)	
Presence of allodynia	18 (43.9 %)	23 (59.0 %)	0.178
Triptan naïve	18 (43.9 %)	24 (61.5 %)	0.176
Current prophylaxis	6 (14.6 %)	15 (28.4 %)	**0.015**
Menopause	7 (17.1 %)	6 (16.2 %)	0.754
Oral contraceptives	4 (9.8 %)	3 (8.1 %)	0.729
Comorbidities	18 (43.9 %)	18 (46.1 %)	0.840

Continuous variables are reported as mean ± SD and categorical variables as frequencies (%)

Statistically significant *P* values (*P* < 0.05) are in bold

*MwA* migraine without aura, *MA* migraine with aura, *UAs* unilateral cranial autonomic symptoms

### Efficacy

**Fig. 2:**
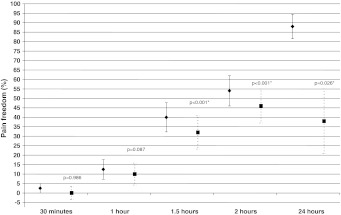
Pain freedom at various time points after oral rizatriptan 10 mg (*diamonds*) intake during an acute migraine attack. *Squares* indicate the therapeutic gain (rizatriptan response–placebo response). *Asterisks* indicate a statistically significant (*P* < 0.05) therapeutic gain

**Fig. 3:**
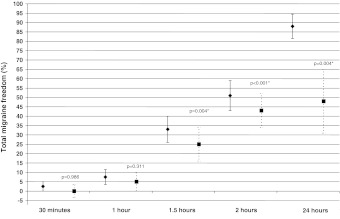
Total migraine freedom at various time points after oral rizatriptan 10 mg (*diamonds*) intake during an acute migraine attack. *Squares* indicate the therapeutic gain (rizatriptan response–placebo response). *Asterisks* indicate a statistically significant (*P* < 0.05) therapeutic gain

Binomial regression analysis showed that a significantly larger percentage of patients assigned to rizatriptan than to placebo reported pain freedom at 2 h post dosing (54 % [95 % CI 38, 70 %] vs. 8 % [95 % CI −1, 17 %]) (*P* < 0.001) (Fig. 2) and total migraine freedom at 2 h post dosing (51 % [95 % CI 36, 67 %] vs. 8 % [95 % CI −1, 17 %]) (*P* < 0.001) (Fig. 3). Active treatment was also more effective than placebo on all the other outcome measures, pain free at 1.5 h, pain relief at, 1.5 and 2 h, no nausea at 2 h, no photophobia at 1.5 and 2 h, 2–24 h sustained pain relief and 2–24 h sustained pain freedom (Table 2). Someother endpoints failed to reach statistical significance after Bonferroni correction, i.e., pain relief at 1 h, total migraine free at 1.5 h, no phonophobia at 1.5–2 h. The recurrence rate was 17.4 % among rizatriptan responders and 25 % among placebo responders. Patients assigned to rizatriptan resorted to rescue medication less frequently than those assigned to placebo (15 vs. 41 %).

**Table 2:** Summary of efficacy data for rizatriptan 10 mg and placebo: primary and secondary endpoints

	Rizatriptan (*n* = 41 pts)	Placebo (*n* = 39 pts)	Therapeutic gain	*P* value
Pain freedom				
0.5 h	1/40 (3 % [−2 %; 7 %])	1/39 (3 % [−2 %; 8 %])	0 % [−7 %; 7 %]	0.986
1 h	5/40 (13 % [2 %; 23 %])	1/39 (3 % [−2 %; 8 %])	10 % [−1 %; 21 %]	0.087
1.5 h	16/40 (40 % [25 %; 55 %])	3/39 (8 % [−1 %; 16 %])	32 % [15 %; 50 %]	<0.001*
2 h	21/39 (54 % [38 %; 70 %])	3/38 (8 % [−1 %; 17 %])	46 % [28 %; 64 %]	<0.001*
Pain relief				
0.5 h	1/40 (3 % [−2 %; 7 %])	1/39 (3 % [−2 %; 8 %])	0 % [−7 %; 7 %]	0.986
1 h	8/40 (20 % [8 %; 32 %])	1/39 (3 % [−2 %; 8 %])	17 % [4 %; 31 %]	**0.010**
1.5 h	20/40 (50 % [35 %; 66 %])	3/39 (8 % [−1 %; 16 %])	42 % [25 %; 60 %]	<0.001*
2 h	23/39 (59 % [44 %; 74 %])	4/38 (11 % [1 %; 20 %])	49 % [30 %; 67 %]	<0.001*
Total migraine freedom				
0.5 h	1/40 (3 % [−2 %; 7 %])	1/39 (3 % [−2 %; 8 %])	0 % [−7 %; 7 %]	0.986
1 h	3/40 (8 % [−1 %; 16 %])	1/39 (3 % [−2 %; 8 %])	5 % [−5 %; 15 %]	0.311
1.5 h	13/40 (33 % [18 %; 47 %])	3/39 (8 % [−1 %; 16 %])	25 % [8 %; 42 %]	**0.004**
2 h	20/39 (51 % [36 %; 67 %])	3/38 (8 % [−1 %; 17 %])	43 % [26 %; 61 %]	<0.001*
No nausea				
0.5 h	3/22 (14 % [−1 %; 28 %])	2/23 (9 % [−3 %; 20 %])	5 % [−14 %; 23 %]	0.599
1 h	8/22 (36 % [16 %; 57 %])	13/23 (13 % [−1 %; 27 %])	23 % [−1 %; 48 %]	0.061
1.5 h	10/22 (46 % [25 %; 66 %])	5/23 (22 % [5 %; 39 %])	24 % [−3 %; 51 %]	0.083
2 h	13/21 (62 % [41 %; 83 %])	3/23 (13 % [−1 %; 27 %])	49 % [24 %; 74 %]	<0.001*
No photophobia				
0.5 h	3/31 (10 % [−1 %; 20 %])	1/25 (4 % [−4 %; 12 %])	6 % [−7 %; 19 %]	0.390
1 h	6/31 (19 % [6 %; 33 %])	1/25 (4 % [−4 %; 12 %])	15 % [−1 %; 31 %]	0.058
1.5 h	14/22 (45 % [28 %; 63 %])	1/25 (4 % [−4 %; 12 %])	41 % [22 %; 60 %]	<0.001*
2 h	16/30 (53 % [36 %; 71 %])	2/24 (8 % [−3 %; 19 %])	45 % [24 %; 66 %]	<0.001*
No phonophobia				
0.5 h	1/29 (4 % [−3 %; 10 %])	2/25 (8 % [−3 %; 19 %])	−5 % [−17 %; 8 %]	0.477
1 h	6/29 (21 % [6 %; 35 %])	2/25 (8 % [−3 %; 19 %])	13 % [−6 %; 31 %]	0.171
1.5 h	13/29 (45 % [27 %; 63 %])	4/25 (16 % [2 %; 30 %])	29 % [6 %; 52 %]	**0.014**
2 h	14/28 (50 % [32 %; 69 %])	4/24 (17 % [2 %; 32 %])	33 % [10 %; 57 %]	**0.006**
2–24 SPF	16/40 (40 % [25 %; 55 %])	1/39 (3 % [−2 %; 8 %])	37 % [22 %; 53 %]	<0.001*
2–24 SPR	18/40 (45 % [30 %; 60 %])	2/39 (5 % [−2 %; 12 %])	40 % [23 %; 57 %]	<0.001*

Data are number of patients (% [95 % confidence interval]). Therapeutic gain = rizatriptan efficacy−placebo efficacy. Statistically significant *P* values (*P* < 0.05) are in bold

*n*, number of treated patients; 2–24 SPF, 2–24 h sustained pain freedom; 2–24 SPR, 2–24 h sustained pain relief

* Type I error <5 % after Bonferroni correction for multiple comparison

### Tolerability and reported adverse events

Although the study primarily investigated efficacy, when we calculated the total number of AEs in each group before patients used rescue medication, the incidence of AEs was similar for rizatriptan and placebo (12 and 10 %) (Table 3). All the AEs were rated as mild.

**Table 3:** Adverse events in both treatment groups

	Rizatriptan 10 mg (*n* = 41 patients)	Placebo (*n* = 39 patients)
Any	5 (12 %)	4 (10 %)
Nausea	1 (2 %)	2 (5 %)
Somnolence	2 (5 %)	0 (0 %)
Dizziness	2 (5 %)	0 (0 %)
Fatigue	0 (0 %)	1 (2.5 %)
Tachycardia	0 (0 %)	1 (2.5 %)

## Discussion

This randomized, double-blind, placebo-controlled parallel-group trial using rizatriptan confirms the results obtained in our previous open study using sumatriptan showing that the presence of UAs in migraineurs predicts highly positive response rates to triptans [5]. Designed as a conventional acute intervention during a moderate-to-severe migraine attack, the present study shows that rizatriptan is consistently more effective than placebo in achieving pain freedom at 2 h and total migraine freedom at 2 h in patients with UAs. Rizatriptan starts to relieve pain 1 h after dosing, achieves pain freedom, total migraine freedom, no photophobia, and no phonophobia at 1.5 h, and eliminates nausea at 2 h. Equally important clinically, rizatriptan is better than placebo for inducing 2–24 h sustained pain relief and 2–24 h sustained pain freedom.

The distinctive finding in our study is the high therapeutic gain for both primary and secondary endpoints. Although our study was not designed to investigate whether migraine patients with UAs respond better to triptans than those without, it offers meaningful evidence-based data on outcomes for comparison. When compared with the therapeutic gain reported in two meta-analyses [10, 11] investigating rizatriptan efficacy in a general migraine population, our study yielded a 15 % absolute increase for 2 h pain freedom (46 vs. 31 %), 15 % for 2 h total migraine freedom (43 vs. 28 %),16 % for 2 h pain relief (49 vs. 33 %), 19 % for 2–24 h sustained pain freedom (37 vs. 18 %), 21 % for 2–24 h sustained pain relief (40 vs. 19 %), 28 % for eliminating nausea (49 vs. 21 %), 17 % for eliminating photophobia (45 vs. 28 %), and 7 % for eliminating phonophobia (33 vs. 26 %). Another interesting finding for clinical and research purposes was that in our patients, all of whom treated their migraine headache only when it became moderate to severe, the therapeutic gain for 2 h pain freedom and 2–24 h sustained pain free (46 and 37 %) almost matched that reported for early treatment (48 and 43 %) [12].

The unexpectedly low placebo effect we found in this study is difficult to explain. Although age is a major variable predicting a placebo effect, patients over 50 years of age being less likely to respond to placebo and more likely to respond to rizatriptan [13], in our study age had no predictive effect because our patients’ mean age was about 40 years. Another possibility is that the low placebo effect and the high rizatriptan response at least partly depended on the fact that many of our patients (61.5 %) were prior triptan users and could therefore discriminate better between placebo and drug (had already experienced triptan-related adverse events) or be triptan responders. Another unanswered question is why the incidence of rizatriptan-related AEs was similar in the treated and placebo groups.

When we screened for UAs in the migraineurs recruited for the study, some reported UAs spontaneously whereas others reported them only after specific questioning. UAs are more frequent in patients with migraine than might be believed. A population-based study using combined postal mail and telephone interviews showed that 26.9 % of migraine patients report at least one of the UAs during their migraine attack regularly, whereas a survey with face-to-face interviews in a headache center discloses UAs in 45.8 % of migraineurs [3, 4]. The presence of UAs depends upon activating the trigeminal autonomic reflex, a physiological response intended to protect ocular and nasal tissue integrity from harmful stimuli. The trigeminal autonomic reflex consists of functional connections between trigeminal afferent fibers and parasympathetic efferents which arise from the superior salivatory nucleus, exit the brainstem via the seventh cranial nerve, traverse the geniculate ganglion and synapse in the sphenopalatine, otic and carotid miniganglia, thereby providing secretomotor innervation to structures such as the lacrimal glands and nasal mucosa [2]. In migraineurs without UAs only the trigeminal afferent arm is active, whereas in patients with UAs the efferent reflex parasympathetic arm is active as well. Migraineurs with UAs experience their headache predominantly on one side, report enhanced pain intensity and have a more frequent facial pain distribution than patients without UAs. Pain severity also correlates weakly with the number of UAs [4, 14]. It is noteworthy that clinical features allow to easily distinguish migraineurs with UAs from patients affected by migraine–cluster headache (a debated syndrome characterized by ‘a headache with predominant symptoms of migraine with at least one major timing factor plus three lesser features of cluster headache, or five lesser features of cluster headache’) and also by trigeminal autonomic cephalgias or sinus pathology [15].

A plausible explanation for our patients’ remarkable response to rizatriptan is that intense trigeminal peripheral afferent activation or sensitization in migraine patients with UAs strongly recruits peripheral neurovascular 5-HT1B/1D receptors, those targeted by triptans. The greater headache severity in these patients presumably reaches the pain threshold above which the autonomic reflex discharges and triggers UAs. This hypothesis receives support also from the clinical finding that patients usually report experiencing UAs when their migraine headache peaks [4]. The precise pathophysiological mechanism underlying trigemino-autonomic reflex activation in migraine is still unclear and could also involve other functional or anatomical peculiarities, or both, in the trigeminal and cranial parasympathetic systems [16].

Our study helps identify a more tailored strategy for treating acute migraine. The search for strategies to improve responsiveness to triptans, given that these anti-migraine drugs fail to achieve good results in many individuals when studies consider strong endpoints (e.g., 2 h pain or migraine freedom), identifies as a crucial issue treatment timing. Treating an attack early widens responsiveness to triptans in allodynic patients by preventing central sensitization from developing [17]. Our findings also emphasize the importance of precisely characterizing the migraine phenotype to predict migraine responses. Here we indicate that simple and relatively common clinical features, namely UAs, may have a positive predictive value in rizatriptan therapy and, probably, in triptan therapy. Whether these observations apply also to other acute or preventive migraine medications merits further research.

Despite its strong point as a randomized, double-blind, placebo-controlled pharmacological trial in migraine patients with UAs, our study has limitations, for example the lack of direct comparison between migraine patients with and without UAs and the fact that we did not enrol only triptan-naïve patients.

In conclusion, migraine patients with UAs, a frequent yet often underdiagnosed category, are a clinically homogeneous migraine population who share a very good response to rizatriptan (and probably to other triptans) even when they use this drug to treat migraine headache that is already moderate or severe. We suggest that pharmacological trials for acute or preventive migraine medications should focus also on this subset of migraine patients.
